# Supplementary material for: Elevated serum YKL-40, IL-6, CRP, CEA, and CA19-9 combined as a prognostic biomarker panel after resection of colorectal liver metastases
Source: PLoS One. 2020 Aug 5;15(8):e0236569. doi: 10.1371/journal.pone.0236569 (PMC7406016; doi:10.1371/journal.pone.0236569)
Supplement: S1 Fig — (DOC) [file pone.0236569.s001.doc]

**Supplementary Figure 1. Study flow diagram.**

Patients undergoing liver resection for colorectal metastases between March 1998 and February 2013

**N=455**

Excluded (n=14)

♦  Extrahepatic metastases (n=7)

♦  Non-radical liver resection (n=6)

♦  Death within 20 days after surgery (n=1)

Patients included in the study

**N=441**
