# Supplementary material for: Elevated serum YKL-40, IL-6, CRP, CEA, and CA19-9 combined as a prognostic biomarker panel after resection of colorectal liver metastases
Source: PLoS One. 2020 Aug 5;15(8):e0236569. doi: 10.1371/journal.pone.0236569 (PMC7406016; doi:10.1371/journal.pone.0236569)

**Supplementary Figure 2. ROC curves describing true positive (TP) and false positive (FP) rates for predicting mortality within 3 years from baseline for a random subpopulation of 25% of the entire cohort.**


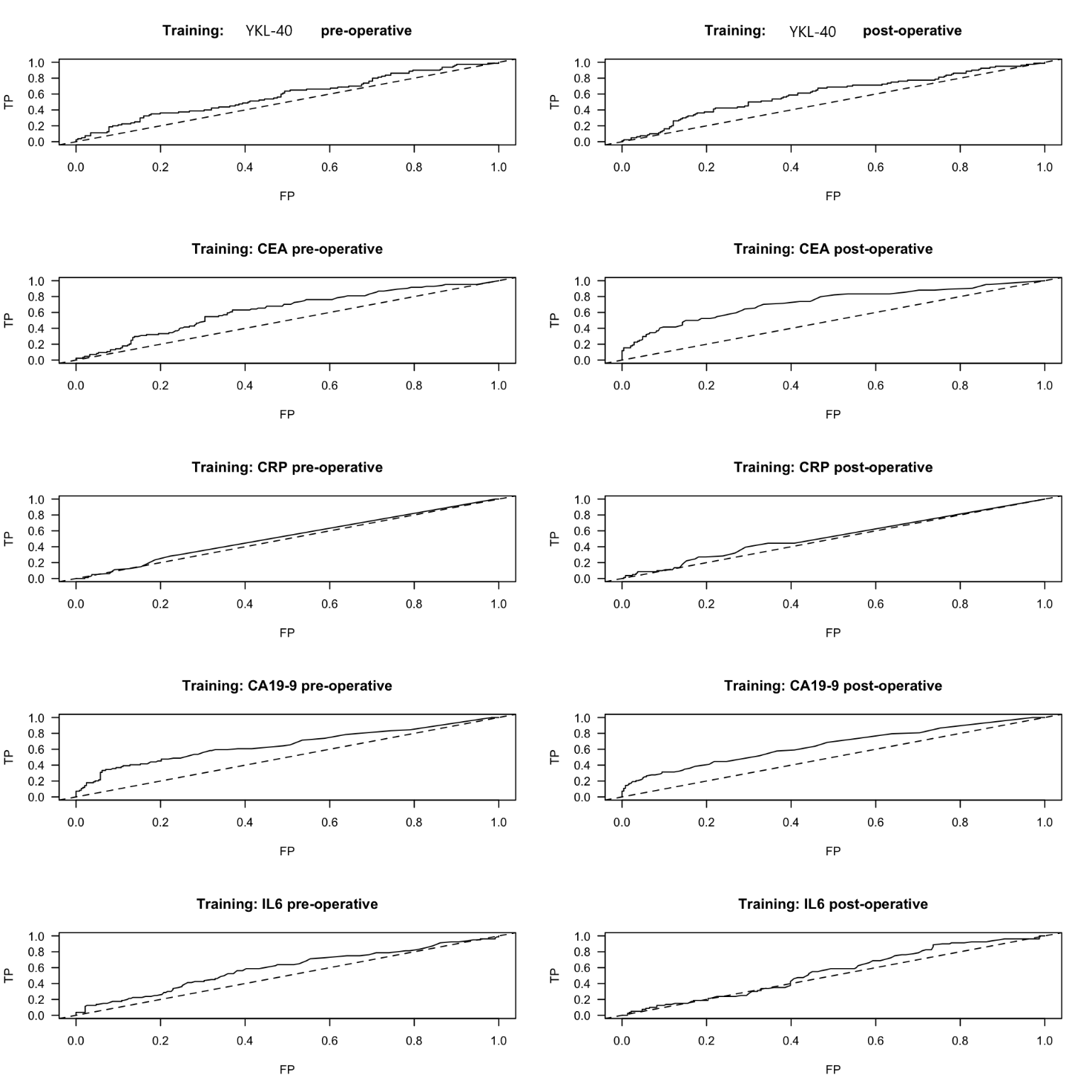

Supplement: S2 Fig — (DOCX) [file pone.0236569.s002.docx]
