# Supplementary material for: Elevated serum YKL-40, IL-6, CRP, CEA, and CA19-9 combined as a prognostic biomarker panel after resection of colorectal liver metastases
Source: PLoS One. 2020 Aug 5;15(8):e0236569. doi: 10.1371/journal.pone.0236569 (PMC7406016; doi:10.1371/journal.pone.0236569)
Supplement: S1 Table — (DOCX) [file pone.0236569.s003.docx]

**Supplementary Table 1. AUC and sensitivity analysis for predicting relapse-free survival 3 years after liver resection for pre-specified cut-off values as defined in the legend.**

| Biomarker | AUC [95 % confidence interval] | Cohort | True positives | False positives | N |
| --- | --- | --- | --- | --- | --- |
| YKL-40^a^ | 62.52 [56.30-68.74] | Validation | 0.13 | 0.13 | 111 |
| YKL-40^a^ |  | Training | 0.19 | 0.07 | 330 |
| YKL-40^b^ | 60.13 [53.84-66.42] | Validation | 0.19 | 0.18 | 111 |
| YKL-40^b^ |  | Training | 0.25 | 0.10 | 330 |
| CEA^a^ | 50.10 [43.73-56.47] | Validation | 0.49 | 0.37 | 111 |
| CEA^a^ |  | Training | 0.51 | 0.55 | 330 |
| CEA^b^ | 62.29 [56.28-68.30] | Validation | 0.13 | 0.12 | 111 |
| CEA^b^ |  | Training | 0.25 | 0.08 | 330 |
| CRP^a^ | 49.85 [44.92-54.78] | Validation | 0.26 | 0.14 | 111 |
| CRP^a^ |  | Training | 0.20 | 0.19 | 330 |
| CRP^b^ | 52.92 [47.27-58.58] | Validation | 0.23 | 0.24 | 111 |
| CRP^b^ |  | Training | 0.36 | 0.25 | 330 |
| CA19-9^a^ | 56.98 [50.81-63.16] | Validation | 0.30 | 0.14 | 111 |
| CA19-9^a^ |  | Training | 0.27 | 0.16 | 330 |
| CA19-9^b^ | 52.80 [46.48-59.12] | Validation | 0.08 | 0.08 | 111 |
| CA19-9^b^ |  | Training | 0.14 | 0.05 | 330 |
| IL-6^a^ | 55.83 [49.34-62.32] | Validation | 0.31 | 0.27 | 111 |
| IL-6^a^ |  | Training | 0.40 | 0.30 | 330 |
| IL-6^b^ | 55.64 [49.15-62.14] | Validation | 0.46 | 0.40 | 111 |
| IL-6^b^ |  | Training | 0.59 | 0.48 | 330 |

An age-corrected 95^th^ percentile for YKL-40 calculated with the formula reported by Bojesen et al [1]; 4.95 pg/ml for IL-6; 5 mg/l for CRP; 5 µg/l for CEA; and 37 kU/l for CA19-9. ^a^Preoperative value; ^b^postoperative value.

[1] Bojesen SE, Johansen JS, Nordestgaard BG: Plasma YKL-40 levels in healthy subjects from the general population. *Clin Chim Acta* 2011, 412(9-10):709-712.
