# Supplementary material for: Elevated serum YKL-40, IL-6, CRP, CEA, and CA19-9 combined as a prognostic biomarker panel after resection of colorectal liver metastases
Source: PLoS One. 2020 Aug 5;15(8):e0236569. doi: 10.1371/journal.pone.0236569 (PMC7406016; doi:10.1371/journal.pone.0236569)
Supplement: S2 Table — The cut-off was optimized as the one closest to providing a sensitivity of 80% for predicting progression-free survival 3 years after liver resection. (DOCX) [file pone.0236569.s004.docx]

**Supplementary Table 2. Cut point analysis for all biomarkers investigated.**

| Biomarker | Cut point | Cohort | True positives | False positives | N |
| --- | --- | --- | --- | --- | --- |
| YKL-40^a^ | 34.8* | Validation | 0.67 | 0.67 | 111 |
| YKL-40^a^ | 34.8* | Training | 0.80 | 0.67 | 330 |
| YKL-40^b^ | 36.5* | Validation | 0.79 | 0.67 | 111 |
| YKL-40^b^ | 36.5* | Training | 0.80 | 0.76 | 330 |
| CEA^a^ | 2.3 µg/L | Validation | 0.78 | 0.73 | 111 |
| CEA^a^ | 2.3 µg/L | Training | 0.80 | 0.78 | 330 |
| CEA^b^ | 1.4 µg/L | Validation | 0.82 | 0.71 | 111 |
| CEA^b^ | 1.4 µg/L | Training | 0.80 | 0.73 | 330 |
| CRP^a^ | 3.0 mg/L | Validation | 1.00 | 1.00 | 111 |
| CRP^a^ | 3.0 mg/L | Training | 0.99 | 1.00 | 330 |
| CRP^b^ | 2.5 mg/L | Validation | 1.00 | 1.00 | 111 |
| CRP^b^ | 2.5 mg/L | Training | 1.00 | 1.00 | 330 |
| CA19-9^a^ | 4.0 kU/L | Validation | 0.85 | 0.74 | 111 |
| CA19-9^a^ | 4.0 kU/L | Training | 0.81 | 0.80 | 330 |
| CA19-9^b^ | 4.0 kU/L | Validation | 0.73 | 0.72 | 111 |
| CA19-9^b^ | 4.0 kU/L | Training | 0.79 | 0.77 | 330 |
| IL-6^a^ | 2.0 pg/mL | Validation | 0.76 | 0.65 | 111 |
| IL-6^a^ | 2.0 pg/mL | Training | 0.80 | 0.79 | 330 |
| IL-6^b^ | 2.9 pg/mL | Validation | 0.75 | 0.58 | 111 |
| IL-6^b^ | 2.9 pg/mL | Training | 0.80 | 0.73 | 330 |

^a^Preoperative value; ^b^postoperative value; *age-corrected percentile value.
