# Supplementary material for: Elevated serum YKL-40, IL-6, CRP, CEA, and CA19-9 combined as a prognostic biomarker panel after resection of colorectal liver metastases
Source: PLoS One. 2020 Aug 5;15(8):e0236569. doi: 10.1371/journal.pone.0236569 (PMC7406016; doi:10.1371/journal.pone.0236569)
Supplement: S3 Table — (DOCX) [file pone.0236569.s005.docx]

**Supplementary Table 3. AUC and sensitivity analysis for predicting mortality 3 years after liver resection for pre-specified cut-off values as defined in the legend.**

| Biomarker | AUC [95 % confidence interval] | Cohort | True positives | False positives | N |
| --- | --- | --- | --- | --- | --- |
| YKL-40^a^ | 58.41 [51.06-65.76] | Validation | 0.17 | 0.12 | 111 |
| YKL-40^a^ |  | Training | 0.23 | 0.11 | 330 |
| YKL-40^b^ | 60.87 [53.55-68.19] | Validation | 0.27 | 0.16 | 111 |
| YKL-40^b^ |  | Training | 0.30 | 0.15 | 330 |
| CEA^a^ | 63.21[56.42-70.00] | Validation | 0.59 | 0.39 | 111 |
| CEA^a^ |  | Training | 0.68 | 0.47 | 330 |
| CEA^b^ | 72.64[65.99-79.29] | Validation | 0.22 | 0.10 | 111 |
| CEA^b^ |  | Training | 0.42 | 0.10 | 330 |
| CRP^a^ | 52.75[47.08-58.43] | Validation | 0.23 | 0.20 | 111 |
| CRP^a^ |  | Training | 0.23 | 0.19 | 330 |
| CRP^b^ | 53.26 [46.56-59.97] | Validation | 0.19 | 0.25 | 111 |
| CRP^b^ |  | Training | 0.40 | 0.29 | 330 |
| CA19-9^a^ | 65.65[58.33-72.97] | Validation | 0.37 | 0.18 | 111 |
| CA19-9^a^ |  | Training | 0.42 | 0.16 | 330 |
| CA19-9^b^ | 65.12[58.04-72.19] | Validation | 0.15 | 0.06 | 111 |
| CA19-9^b^ |  | Training | 0.25 | 0.05 | 330 |
| IL-6^a^ | 58.84[51.41-66.27] | Validation | 0.25 | 0.31 | 111 |
| IL-6^a^ |  | Training | 0.45 | 0.33 | 330 |
| IL-6^b^ | 54.55[47.55-61.54] | Validation | 0.38 | 0.45 | 111 |
| IL-6^b^ |  | Training | 0.59 | 0.53 | 330 |

An age-corrected 95^th^ percentile for serum YKL-40 calculated with the formula reported by Bojesen et al [1]; 4.95 pg/ml for IL-6; 5 mg/l for CRP; 5 µg/l for CEA; and 37 kU/l for CA19-9. ^a^Preoperative value; ^b^postoperative value.

[1] Bojesen SE, Johansen JS, Nordestgaard BG: Plasma YKL-40 levels in healthy subjects from the general population. *Clin Chim Acta* 2011, 412(9-10):709-712.
