# Supplementary material for: Elevated serum YKL-40, IL-6, CRP, CEA, and CA19-9 combined as a prognostic biomarker panel after resection of colorectal liver metastases
Source: PLoS One. 2020 Aug 5;15(8):e0236569. doi: 10.1371/journal.pone.0236569 (PMC7406016; doi:10.1371/journal.pone.0236569)
Supplement: S4 Table — The cut-off was optimized as the one closest to providing a sensitivity of 80% for predicting mortality 3 years after liver resection. (DOCX) [file pone.0236569.s006.docx]

**Supplementary Table 4. Cut point analysis for all biomarkers investigated.**

| Biomarker | Cut point | Cohort | True positives | False positives | N |
| --- | --- | --- | --- | --- | --- |
| YKL-40^a^ | 36.4^*^ | Validation | 0.63 | 0.65 | 111 |
| YKL-40^a^ | 36.4^*^ | Training | 0.80 | 0.71 | 330 |
| YKL-40^b^ | 38.0^*^ | Validation | 0.73 | 0.71 | 111 |
| YKL-40^b^ | 38.0^*^ | Training | 0.80 | 0.75 | 330 |
| CEA^a^ | 3.4 µg/L | Validation | 0.78 | 0.54 | 111 |
| CEA^a^ | 3.4 µg/L | Training | 0.80 | 0.63 | 330 |
| CEA^b^ | 2.3 µg/L | Validation | 0.63 | 0.46 | 111 |
| CEA^b^ | 2.3 µg/L | Training | 0.80 | 0.47 | 330 |
| CRP^a^ | 1.0 mg/L | Validation | 1.00 | 1.00 | 111 |
| CRP^a^ | 1.0 mg/L | Training | 1.00 | 1.00 | 330 |
| CRP^b^ | 2.5 mg/L | Validation | 1.00 | 1.00 | 111 |
| CRP^b^ | 2.5 mg/L | Training | 1.00 | 1.00 | 330 |
| CA19-9^a^ | 6.0 kU/L | Validation | 0.93 | 0.64 | 111 |
| CA19-9^a^ | 6.0 kU/L | Training | 0.81 | 0.69 | 330 |
| CA19-9^b^ | 6.0 kU/L | Validation | 0.81 | 0.57 | 111 |
| CA19-9^b^ | 6.0 kU/L | Training | 0.80 | 0.64 | 330 |
| IL-6^a^ | 2.0 pg/mL | Validation | 0.75 | 0.69 | 111 |
| IL-6^a^ | 2.0 pg/mL | Training | 0.81 | 0.79 | 330 |
| IL-6^b^ | 3.3 pg/mL | Validation | 0.69 | 0.59 | 111 |
| IL-6^b^ | 3.3 pg/mL | Training | 0.81 | 0.71 | 330 |

^a^Preoperative value; ^b^postoperative value; *age-corrected percentile value.
