# Supplementary material for: Elevated serum YKL-40, IL-6, CRP, CEA, and CA19-9 combined as a prognostic biomarker panel after resection of colorectal liver metastases
Source: PLoS One. 2020 Aug 5;15(8):e0236569. doi: 10.1371/journal.pone.0236569 (PMC7406016; doi:10.1371/journal.pone.0236569)
Supplement: S5 Table — (A-D) Cox regression analysis estimates for all variables for both pre- and postoperative biomarker values and associations with (A and B) overall survival and (C and D) relapse-free survival. (DOCX) [file pone.0236569.s007.docx]

| **Supplementary Table 5 A. Multivariate HRs for OS (preoperative biomarkers).** | | | | | |  |
| --- | --- | --- | --- | --- | --- | --- |
| **Variable** | **Units** | **Missing** | **Hazard Ratio** | **95% CI** | **p-value** | |
| Primary tumor location | Rectum | 0 | Ref |  |  | |
|  | Left colon |  | 0.75 | [0.56–0.99] | 0.042 | |
|  | Right colon |  | 0.65 | [0.45–0.96] | 0.031 | |
| Synchronous / metachronous metastases | 0 | 0 | Ref |  |  | |
|  | 1 |  | 1.04 | [0.78–1.39] | 0.767 | |
| Number of liver metastases | 0-2 | 0 | Ref |  |  | |
|  | 3-5 |  | 1.47 | [1.03–2.10] | 0.033 | |
|  | 6+ |  | 1.93 | [1.01–3.70] | 0.048 | |
| Metastasis size above 6 mm | FALSE | 0 | Ref |  |  | |
|  | TRUE |  | 1.16 | [0.68–1.99] | 0.593 | |
| Operation type | major | 1 | Ref |  |  | |
|  | minor |  | 0.97 | [0.72–1.33] | 0.864 | |
| Gender | Male | 0 | Ref |  |  | |
|  | Female |  | 0.96 | [0.73–1.26] | 0.757 | |
| Age |  | 0 | 1.02 | [1.01–1.04] | <0.001 | |
| Radicality | 0 | 1 | Ref |  |  | |
|  | 1 |  | 1.32 | [0.76–2.28] | 0.329 | |
|  | 2 |  | 2.01 | [0.79–5.16] | 0.145 | |
| IL-6_pre_log2 |  | 28 | 1.03 | [0.90–1.18] | 0.685 | |
| YKL-40_pre_log2 |  | 28 | 1.19 | [1.04–1.35] | 0.010 | |
| CRP_pre_log2 |  | 12 | 0.91 | [0.77–1.06] | 0.215 | |
| CEA_pre_log2 |  | 1 | 1.02 | [0.95–1.09] | 0.602 | |
| CA19-9_pre_log2 |  | 4 | 1.13 | [1.06–1.20] | <0.001 | |
|  |  |  |  |  |  | |

| **Supplementary Table 5 B. Multivariate HRs for OS (postoperative biomarkers).** | | | | | |
| --- | --- | --- | --- | --- | --- |
| **Variable** | **Units** | **Missing** | **Hazard Ratio** | **95% CI** | **p-value** |
| Primary tumor location | Rectum | 0 | Ref |  |  |
|  | Left colon |  | 0.84 | [0.63–1.11] | 0.224 |
|  | Right colon |  | 0.78 | [0.54–1.13] | 0.184 |
| Synchronous / metachronous metastases | 0 | 0 | Ref |  |  |
|  | 1 |  | 1.08 | [0.83–1.40] | 0.589 |
| Number of liver metastases | 0-2 | 0 | Ref |  |  |
|  | 3-5 |  | 1.68 | [1.18–2.39] | 0.004 |
|  | 6+ |  | 1.79 | [0.94–3.41] | 0.075 |
| Metastasis size above 6 mm | FALSE | 0 | Ref |  |  |
|  | TRUE |  | 1.74 | [1.08–2.80] | 0.024 |
| Operation type | major | 1 | Ref |  |  |
|  | minor |  | 0.89 | [0.65–1.22] | 0.485 |
| Gender | Male | 0 | Ref |  |  |
|  | Female |  | 1.05 | [0.80–1.38] | 0.711 |
| Age |  | 0 | 1.03 | [1.01–1.04] | <0.001 |
| Radicality | 0 | 1 | Ref |  |  |
|  | 1 |  | 1.38 | [0.78–2.46] | 0.272 |
|  | 2 |  | 1.88 | [0.79–4.47] | 0.155 |
| IL-6_post_log2 |  | 28 | 0.95 | [0.83–1.09] | 0.498 |
| YKL-40_post_log2 |  | 28 | 1.10 | [0.97–1.24] | 0.131 |
| CRP_post_log2 |  | 7 | 1.09 | [0.99–1.20] | 0.096 |
| CEA_post_log2 |  | 1 | 1.24 | [1.12–1.36] | <0.001 |
| CA19-9_post_log2 |  | 5 | 1.17 | [1.08–1.28] | <0.001 |

| **Supplementary Table 5 C. Multivariate HRs for RFS (preoperative biomarkers)** | | | | | |
| --- | --- | --- | --- | --- | --- |
| **Variable** | **Units** | **Missing** | **Hazard Ratio** | **95% CI** | **p-value** |
| Primary tumor location | Rectum | 0 | Ref |  |  |
|  | Left colon |  | 0.73 | [0.56–0.95] | 0.018 |
|  | Right colon |  | 0.58 | [0.40–0.83] | 0.003 |
| Synchronous / metachronous metastases | 0 | 0 | Ref |  |  |
|  | 1 |  | 1.42 | [1.08–1.86] | 0.013 |
| Number of liver metastases | 0-2 | 0 | Ref |  |  |
|  | 3-5 |  | 1.60 | [1.16–2.22] | 0.004 |
|  | 6+ |  | 4.24 | [2.33–7.69] | <0.001 |
| Metastasis size above 6 mm | FALSE | 0 | Ref |  |  |
|  | TRUE |  | 0.89 | [0.53–1.49] | 0.650 |
| Operation type | major | 1 | Ref |  |  |
|  | minor |  | 1.15 | [0.87–1.53] | 0.335 |
| Gender | Male | 0 | Ref |  |  |
|  | Female |  | 1.02 | [0.79–1.32] | 0.889 |
| Age |  | 0 | 1.02 | [1.01–1.04] | 0.001 |
| Radicality | 0 | 1 | Ref |  |  |
|  | 1 |  | 1.51 | [0.90–2.54] | 0.120 |
|  | 2 |  | 3.26 | [1.37–7.80] | 0.008 |
| IL-6_pre_log2 |  | 28 | 1.04 | [0.91–1.18] | 0.566 |
| YKL-40_pre_log2 |  | 28 | 1.08 | [0.96–1.22] | 0.212 |
| CRP_pre_log2 |  | 12 | 1.03 | [0.88–1.20] | 0.756 |
| CEA_pre_log2 |  | 1 | 1.00 | [0.93–1.07] | 0.952 |
| CA19-9_pre_log2 |  | 4 | 1.12 | [1.06–1.19] | <0.001 |

| **Supplementary Table 5 D. Multivariate HRs for RFS (postoperative biomarkers).** | | | | | |
| --- | --- | --- | --- | --- | --- |
| **Variable** | **Units** | **Missing** | **Hazard Ratio** | **95% CI** | **p-value** |
| Primary tumor location | Rectum | 0 | Ref |  |  |
|  | Left colon |  | 0.84 | [0.64–1.10] | 0.200 |
|  | Right colon |  | 0.64 | [0.45–0.90] | 0.011 |
| Synchronous / metachronous metastases | 0 | 0 | Ref |  |  |
|  | 1 |  | 1.54 | [1.20–1.98] | <0.001 |
| Number of liver metastases | 0-2 | 0 | Ref |  |  |
|  | 3-5 |  | 1.85 | [1.34–2.55] | <0.001 |
|  | 6+ |  | 3.77 | [2.11–6.76] | <0.001 |
| Metastasis size above 6 mm | FALSE | 0 | Ref |  |  |
|  | TRUE |  | 1.56 | [0.97–2.48] | 0.065 |
| Operation type | major | 1 | Ref |  |  |
|  | minor |  | 1.18 | [0.88–1.58] | 0.276 |
| Gender | Male | 0 | Ref |  |  |
|  | Female |  | 1.06 | [0.83–1.36] | 0.639 |
| Age |  | 0 | 1.03 | [1.02–1.04] | <0.001 |
| Radicality | 0 | 1 | Ref |  |  |
|  | 1 |  | 1.44 | [0.85–2.43] | 0.171 |
|  | 2 |  | 2.42 | [1.09–5.39] | 0.030 |
| IL-6_post_log2 |  | 28 | 0.98 | [0.87–1.11] | 0.755 |
| YKL-40_post_log2 |  | 28 | 1.06 | [0.94–1.19] | 0.323 |
| CRP_post_log2 |  | 7 | 1.02 | [0.92–1.12] | 0.741 |
| CEA_post_log2 |  | 1 | 1.17 | [1.07–1.29] | <0.001 |
| CA19-9_post_log2 |  | 5 | 1.09 | [1.01–1.17] | 0.029 |
