# Supplementary material for: Elevated serum YKL-40, IL-6, CRP, CEA, and CA19-9 combined as a prognostic biomarker panel after resection of colorectal liver metastases
Source: PLoS One. 2020 Aug 5;15(8):e0236569. doi: 10.1371/journal.pone.0236569 (PMC7406016; doi:10.1371/journal.pone.0236569)
Supplement: S6 Table — (DOCX) [file pone.0236569.s008.docx]

| **Multivariate HRs for RFS (preoperative).** | | | | | |
| --- | --- | --- | --- | --- | --- |
| **Variable** | **Units** | **Missing** | **Hazard Ratio** | **95% CI** | **p-value** |
| Primary tumor location | Rectum | 0 | Ref |  |  |
|  | Left colon |  | 0.72 | [0.56;0.94] | 0.016 |
|  | Right colon |  | 0.58 | [0.41;0.83] | 0.003 |
| Synchronous / metachronous metastases | 0 | 0 | Ref |  |  |
|  | 1 |  | 1.28 | [0.98;1.67] | 0.065 |
| Number of liver metastases | 0-2 | 0 | Ref |  |  |
|  | 3-5 |  | 1.63 | [1.18;2.26] | 0.003 |
|  | 6+ |  | 4.36 | [2.42;7.83] | <0.001 |
| Metastasis size above 6 mm | FALSE | 0 | Ref |  |  |
|  | TRUE |  | 1.03 | [0.65;1.64] | 0.890 |
| Operation type | major | 1 | Ref |  |  |
|  | minor |  | 1.09 | [0.82;1.45] | 0.549 |
| Gender | Male | 0 | Ref |  |  |
|  | Female |  | 1.00 | [0.78;1.29] | 0.995 |
| Age |  | 0 | 1.02 | [1.01;1.04] | <0.001 |
| Radicality | 0 | 1 | Ref |  |  |
|  | 1 |  | 1.45 | [0.86;2.42] | 0.161 |
|  | 2 |  | 3.50 | [1.50;8.19] | 0.004 |
| NumberOfElevatedprecat | 0 | 42 | Ref |  |  |
|  | CA19-9 or CEA elevated |  | 1.16 | [0.87;1.54] | 0.328 |
|  | IL6 or YKL40 or CRP |  | 0.92 | [0.64;1.33] | 0.671 |

| **Multivariate HRs for RFS (postoperative).** | | | | | |
| --- | --- | --- | --- | --- | --- |
| **Variable** | **Units** | **Missing** | **Hazard Ratio** | **95% CI** | **p-value** |
| Primary tumor location | Rectum | 0 | Ref |  |  |
|  | Left colon |  | 0.82 | [0.63;1.06] | 0.130 |
|  | Right colon |  | 0.64 | [0.45;0.90] | 0.010 |
| Synchronous / metachronous metastases | 0 | 0 | Ref |  |  |
|  | 1 |  | 1.50 | [1.17;1.93] | 0.001 |
| Number of liver metastases | 0-2 | 0 | Ref |  |  |
|  | 3-5 |  | 1.85 | [1.34;2.56] | <0.001 |
|  | 6+ |  | 3.80 | [2.12;6.83] | <0.001 |
| Metastasis size above 6 mm | FALSE | 0 | Ref |  |  |
|  | TRUE |  | 1.55 | [0.98;2.45] | 0.062 |
| Operation type | major | 1 | Ref |  |  |
|  | minor |  | 1.15 | [0.86;1.53] | 0.342 |
| Gender | Male | 0 | Ref |  |  |
|  | Female |  | 1.03 | [0.80;1.32] | 0.844 |
| Age |  | 0 | 1.03 | [1.02;1.05] | <0.001 |
| Radicality | 0 | 1 | Ref |  |  |
|  | 1 |  | 1.28 | [0.77;2.15] | 0.342 |
|  | 2 |  | 2.73 | [1.24;5.98] | 0.012 |
| NumberOfElevatedpostcat | 0 | 38 | Ref |  |  |
|  | CA19-9 or CEA |  | 1.87 | [1.36;2.58] | <0.001 |
|  | IL6 or YKL40 or CRP |  | 1.07 | [0.80;1.41] | 0.658 |

| **Multivariate HRs for OS (preoperative).** | | | | | |
| --- | --- | --- | --- | --- | --- |
| **Variable** | **Units** | **Missing** | **Hazard Ratio** | **95% CI** | **p-value** |
| Primary tumor location | Rectum | 0 | Ref |  |  |
|  | Left colon |  | 0.75 | [0.56;0.99] | 0.043 |
|  | Right colon |  | 0.65 | [0.44;0.96] | 0.028 |
| Synchronous / metachronous metastases | 0 | 0 | Ref |  |  |
|  | 1 |  | 0.98 | [0.75;1.30] | 0.911 |
| Number of liver metastases | 0-2 | 0 | Ref |  |  |
|  | 3-5 |  | 1.45 | [1.02;2.07] | 0.041 |
|  | 6+ |  | 1.82 | [0.96;3.44] | 0.068 |
| Metastasis size above 6 mm | FALSE | 0 | Ref |  |  |
|  | TRUE |  | 1.23 | [0.76;1.98] | 0.400 |
| Operation type | major | 1 | Ref |  |  |
|  | minor |  | 0.91 | [0.67;1.23] | 0.531 |
| Gender | Male | 0 | Ref |  |  |
|  | Female |  | 0.99 | [0.76;1.30] | 0.959 |
| Age |  | 0 | 1.03 | [1.01;1.04] | <0.001 |
| Radicality | 0 | 1 | Ref |  |  |
|  | 1 |  | 1.34 | [0.77;2.32] | 0.300 |
|  | 2 |  | 2.18 | [0.87;5.50] | 0.098 |
| NumberOfElevatedprecat | 0 | 42 | Ref |  |  |
|  | CA19-9 or CEA elevated |  | 1.27 | [0.93;1.74] | 0.134 |
|  | IL6 or YKL40 or CRP |  | 1.03 | [0.69;1.54] | 0.878 |

| **Multivariate HRs for OS (postoperative).** | | | | | |
| --- | --- | --- | --- | --- | --- |
| **Variable** | **Units** | **Missing** | **Hazard Ratio** | **95% CI** | **p-value** |
| Primary tumor location | Rectum | 0 | Ref |  |  |
|  | Left colon |  | 0.78 | [0.59;1.04] | 0.094 |
|  | Right colon |  | 0.76 | [0.53;1.10] | 0.143 |
| Synchronous / metachronous metastases | 0 | 0 | Ref |  |  |
|  | 1 |  | 1.05 | [0.81;1.37] | 0.707 |
| Number of liver metastases | 0-2 | 0 | Ref |  |  |
|  | 3-5 |  | 1.61 | [1.13;2.28] | 0.008 |
|  | 6+ |  | 1.72 | [0.90;3.27] | 0.100 |
| Metastasis size above 6 mm | FALSE | 0 | Ref |  |  |
|  | TRUE |  | 1.63 | [1.02;2.63] | 0.043 |
| Operation type | major | 1 | Ref |  |  |
|  | minor |  | 0.90 | [0.66;1.22] | 0.480 |
| Gender | Male | 0 | Ref |  |  |
|  | Female |  | 1.01 | [0.77;1.33] | 0.926 |
| Age |  | 0 | 1.03 | [1.02;1.04] | <0.001 |
| Radicality | 0 | 1 | Ref |  |  |
|  | 1 |  | 1.16 | [0.66;2.05] | 0.606 |
|  | 2 |  | 1.94 | [0.82;4.56] | 0.129 |
| NumberOfElevatedpostcat | 0 | 38 | Ref |  |  |
|  | CA19-9 or CEA |  | 2.15 | [1.52;3.02] | <0.001 |
|  | IL6 or YKL40 or CRP |  | 1.07 | [0.78;1.47] | 0.662 |
