# Supplementary material for: Elevated serum YKL-40, IL-6, CRP, CEA, and CA19-9 combined as a prognostic biomarker panel after resection of colorectal liver metastases
Source: PLoS One. 2020 Aug 5;15(8):e0236569. doi: 10.1371/journal.pone.0236569 (PMC7406016; doi:10.1371/journal.pone.0236569)
Supplement: Attachment — Submitted filename: Response to Reviewers - PLOS ONE 2020.docx [file pone.0236569.s010.docx]

PLOS ONE

Date: February 28^th^, 2020

**Responses to the comments of the Academic Editor and the Reviewers**

We thank the Academic Editor and the Reviewers for thorough and accurate comments and constructive criticism. We have revised the manuscript according to the suggestions and responded to the comments point by point (please see below). Our responses are written in red italics and the selected quotes from the manuscript text in bold red italics. We strongly feel that these changes have improved the manuscript.

**Journal Requirements:**

When submitting your revision, we need you to address these additional requirements.

1.  Please ensure that your manuscript meets PLOS ONE's style requirements, including those for file naming. The PLOS ONE style templates can be found at <http://www.journals.plos.org/plosone/s/file?id=wjVg/PLOSOne_formatting_sample_main_body.pdf> and <http://www.journals.plos.org/plosone/s/file?id=ba62/PLOSOne_formatting_sample_title_authors_affiliations.pdf>

*We have edited our manuscript, where necessary, to ensure that it meets the style requirements.*

2. Please provide additional details regarding participant consent. In the ethics statement in the Methods and online submission information, please ensure that you have specified whether consent was written or verbal/oral. If consent was verbal/oral, please specify: 1) whether the ethics committee approved the verbal/oral consent procedure, 2) why written consent could not be obtained, and 3) how verbal/oral consent was recorded. If your study included minors, please state whether you obtained consent from parents or guardians in these cases. If the need for consent was waived by the ethics committee, please include this information.

*We have added these details to our manuscript and online submission information as follows:*

*1) The Ethics Committee at Helsinki University Hospital has approved the verbal consent procedure.*

*2)* *The collection of blood samples was initiated over twenty years ago, and at that time, verbal consent was generally accepted. Many of the patients included in our study have already died, and the permission to use the samples in recent studies has been given by the National Supervisory Authority for Welfare and Health (Valvira) of Finland.*

*3) The verbal consent was taken by the treating physician(s) and passed on to the nurse in charge of ordering all the necessary laboratory specimens.* *The collection of the research samples signified participant consent.*

*4) No minors were included in this study.*

3. We note that you have indicated that data from this study are available upon request. PLOS only allows data to be available upon request if there are legal or ethical restrictions on sharing data publicly. For information on unacceptable data access restrictions, please see <http://journals.plos.org/plosone/s/data-availability#loc-unacceptable-data-access-restrictions>.

In your revised cover letter, please address the following prompts:

a) If there are ethical or legal restrictions on sharing a de-identified data set, please explain them in detail (e.g., data contain potentially identifying or sensitive patient information) and who has imposed them (e.g., an ethics committee). Please also provide contact information for a data access committee, ethics committee, or other institutional body to which data requests may be sent.

b) If there are no restrictions, please upload the minimal anonymized data set necessary to replicate your study findings as either Supporting Information files or to a stable, public repository and provide us with the relevant URLs, DOIs, or accession numbers. Please see <http://www.bmj.com/content/340/bmj.c181.long> for guidelines on how to de-identify and prepare clinical data for publication. For a list of acceptable repositories, please see <http://journals.plos.org/plosone/s/data-availability#loc-recommended-repositories>.

*We have now uploaded a fully anonymized data set as a Supporting Information file, as requested.*

**Reviewers' comments:**

Reviewer's Responses to Questions

**Comments to the Author**

1. Is the manuscript technically sound, and do the data support the conclusions?

The manuscript must describe a technically sound piece of scientific research with data that supports the conclusions. Experiments must have been conducted rigorously, with appropriate controls, replication, and sample sizes. The conclusions must be drawn appropriately based on the data presented.

Reviewer #1: No

Reviewer #2: Yes

2. Has the statistical analysis been performed appropriately and rigorously?

Reviewer #1: Yes

Reviewer #2: Yes

3. Have the authors made all data underlying the findings in their manuscript fully available?

The [PLOS Data policy](http://www.plosone.org/static/policies.action#sharing) requires authors to make all data underlying the findings described in their manuscript fully available without restriction, with rare exception (please refer to the Data Availability Statement in the manuscript PDF file). The data should be provided as part of the manuscript or its supporting information, or deposited to a public repository. For example, in addition to summary statistics, the data points behind means, medians and variance measures should be available. If there are restrictions on publicly sharing data—e.g. participant privacy or use of data from a third party—those must be specified.

Reviewer #1: Yes

Reviewer #2: No

4. Is the manuscript presented in an intelligible fashion and written in standard English?

PLOS ONE does not copyedit accepted manuscripts, so the language in submitted articles must be clear, correct, and unambiguous. Any typographical or grammatical errors should be corrected at revision, so please note any specific errors here.

Reviewer #1: Yes

Reviewer #2: Yes

**5. Review Comments to the Author**

Please use the space provided to explain your answers to the questions above. You may also include additional comments for the author, including concerns about dual publication, research ethics, or publication ethics. (Please upload your review as an attachment if it exceeds 20,000 characters)

Reviewer #1: The authors here present data from an impressively large number of patients who underwent resection of liver metastases for their colorectal cancer. They examine the role of 5 biomarkers and conclude elevated expression of a combination of these biomarkers associate with worse survival outcomes. These findings are consistent with prior efforts and fit into the general understanding of clinical management of metastatic colorectal cancer. I think that the message could be strengthened with additional points to be addressed:

1. None of the markers for systemic inflammation are associated with clinical outcomes as a single marker in the multivariate analysis, and the significance is seen only when analyzed "in combination." It is unclear which markers, when "2 or more" were elevated, were actually elevated. If one is to translate these to the clinic, should he or she order all five, or were there specific ones which seemed to be more associated with outcome? As presented, the applicability here is very difficult for the reader.

*We thank the Reviewer for this comment. We have now explored this further by studying three groups of patients in an additional explorative analysis, namely patients with zero elevated markers, patients with CEA and/or CA19-9 elevated, and patients with YKL-40, IL-6 and/or CRP elevated. The results of this analysis are included as S6 Table in the Supporting Information files. They showed that patients with elevated CEA and/or CA19-9 elevated postoperatively had a significantly higher risk of relapse or death, while elevated YKL-40, IL-6, and/or CRP was not significantly associated with increased risk of relapse or death in any instance. We have already analyzed the biomarkers together in a multivariable analysis, although on a continuous scale, which showed that mainly CEA and CA19-9 were the drivers behind the negative prognostic value of having several elevated biomarkers, although YKL-40 was also associated with a negative prognostic outcome as a preoperative marker in relation to OS.*

*We still conclude that the risk of relapse and death associated with an increase in the number of elevated markers, although unspecific, could be due to an additive effect of the markers, each highlighting a different aspect of disease relapse or imminent death. The sections “Results” and “Discussion” have been updated with comments on these explorative findings.*

2. The authors mention that these findings support the use of these markers as a predictive biomarker and "might identify patients who benefit from more adjuvant chemotherapy." However, while there analyses may support the combination of markers PROGNOSTICALLY, the authors provide zero detail of receipt of any adjuvant chemotherapy for these patients. Therefore these claims are unsupported and should be removed as presented from the manuscript.

*We thank the Reviewer for this accurate observation. The manuscript text has been modified as follows (section “Abstract”):*

*“****The results suggest that a protein panel of the inflammatory biomarkers YKL-40, IL-6, and CRP, and the cancer biomarkers CEA and CA19-9 might identify patients that benefit from more aggressive treatment and surveillance.****”*

3. One could argue that if this combination of markers is associated with a poor prognosis, the biology of the given CRC tumor is especially poor, and these might identify the patients who undergo resection that will have a higher risk of relapse and therefore NOT benefit from more surgery if at especially high risk for relapse and shortened OS. I disagree that re-resection would be warranted based on the data presented.

*We fully agree with the Reviewer. We have not included the information on possible re-resections in our data, and thus, their role cannot be evaluated based on our findings. Therefore, we have removed the claim in question from the manuscript and edited the text as follows:*

*Section “Abstract”: “****The results suggest that a protein panel of the inflammatory biomarkers YKL-40, IL-6, and CRP, and the cancer biomarkers CEA and CA19-9 might identify patients that benefit from more aggressive treatment and surveillance.****”*

*Section “Conclusions”: “****In the future, this panel might be used to select the patients that could benefit from more aggressive perioperative chemotherapy and follow-up.****”*

4. If IL-6 is responsible for producing CRP and YKL-40, then if IL-6 is elevated, should the other downstream markers be as well? If so, what is the reason for needed to test all three? IL-6 alone did not identify high-risk patients in the multivariate survival analysis. The authors could consider addressing this in their discussion.

*We thank the reviewer for this thoughtful question. It is true that IL-6 primarily stimulates the production of CRP in the liver, but YKL-40 is also stimulated by other biological processes. Since the studied cohort has metastases to the liver, we must assume some dysfunction of the hepatic production of CRP. This makes studying all parameters in this particular cohort interesting. We have updated the section “Discussion” addressing this aspect in a more elaborate manner.*

5. Clarification of "major" and "minor" hepatic resections (how are these defined?) is needed in the methods section.

*We have defined the meaning of “minor” and “major” 1) in Materials and Methods, in the section “Patients” (“****The liver resections were considered major if more than two Couinaud segments were resected.****”), and 2) in Table 1 (“****^2^ 1–2 (minor) or ≥3 (major) Couinaud segments resected****”).*

6. YKL-40, IL-6 both increased from the preoperative to postoperative setting. One would expect that if these were associated with cancer only, there would be some decrease in median levels following resection, unless most patients recurred in the time before the first postoperative blood specimen was drawn. How do the authors interpret the fact that %patients with elevated inflammatory markers rose after surgery? Could these nonspecific markers be reflective of other factors?

*We thank the Reviewer for this interesting reflection. It is possible that some of the increase is explained by the surgical trauma, although the publication that we have as a reference in the Discussion suggests that this increase wanes after approximately 3 weeks. That is why we attribute – at least in part – the increase to micro-metastatic disease, although we acknowledge that this is partially only speculative.*

*We have now addressed the Reviewer’s questions in the section “Discussion” as follows:*

*“****In addition, serum YKL-40 has been shown to be increased until the 21^st^ postoperative day merely due to the surgical trauma related to primary tumor resection [34].*** ***We sampled blood a median of 94 (IQR 89–98) days after surgery. Thus, the postoperative increase seen in serum YKL-40 and IL-6 in some of the patients could be caused by micro-metastatic disease rather than the large surgical trauma in the liver, although it is possible that some effect of the surgical trauma persisted. We assume that the postoperative changes in inflammatory biomarkers would have normalized before the postoperative sampling.****”*

7. Without knowing which markers were elevated (2 of 5, 3 of 5 -which ones???), the clinical applicability of these findings is low, especially since, as the authors mention, these markers can be attributed to other cancers, autoimmune diseases, etc. The cost effectiveness of this approach is unclear, and the omission of alternate emerging approaches as a prognostic/predictive biomarker such as ctDNA in this setting by the authors is glaring. I am not sure the conclusions from the authors are supported by their data.

*We thank the Reviewer for this comment. We have now defined, which biomarkers were elevated and thus, which combinations were prognostic.*

*In our data, all the patients had only colorectal cancer, and other malignancies were an exclusion criterion. We have now mentioned this in the section “Patients” (“****All patients included were diagnosed with resectable CRLM, and those with possible other malignancies were excluded.****”). Our results suggest that the biomarkers are prognostic in colorectal cancer, but we cannot generalize this to apply to other cancers.*

*We have not included the information concerning possible autoimmune diseases in the database, because there were only a few – if any – patients with those diseases. We assume that if the increase in the biomarker levels would be related to autoimmune diseases instead of colorectal cancer, the number of patients with those diseases should be considerable to confound the biomarker-mortality association.*

*Concerning ctDNA, its role has by far been equivocal, and further research is needed. As the Reviewer suggests, it would have been interesting to investigate ctDNA in our material, but unfortunately, our samples do not fulfill the technical criteria for these analyses. We have now updated the section “Discussion” including ctDNA as an alternate approach in this field.*

Reviewer #2: I would like the commend the authors on a extremely well written manuscript which seeks to evaluate whether elevated serum biomarker levels of YKL-40, IL-6, and CRP may improve upon prognostic significance beyond CEA and CA 19-9. The authors perform a retrospective evaluation of biomarker study of 441 consecutive patients undergoing resection for colorectal liver metastases, where the collected samples prospectively preoperatively and postoperatively. With a median follow-up of 5+ years, the authors found that patients with 2-5 biomarkers elevated were at increased risk of relapse and worse OS compared to those with 0-1 biomarkers elevated. This is a large cohort of patients with a well defined population of patients, with potential for important prognostic and therapeutic implications of treatment. I would ask the authors to clarify the following points to further strengthen their manuscript:

1) Given the awareness and prognostic significance of mutational status, as the authors acknowledged, this manuscript would be further improved if mutational status were available to correlate with inflammatory biomarkers. Was microsatellite status available on any of these patients?

*We fully agree with the Reviewer. Unfortunately, the microsatellite status was not routinely analyzed at our hospital prior to 2018. We have now specified this in the manuscript text as follows (section “Discussion”):*

*“****Information about the KRAS, NRAS and BRAF mutational status as well as the microsatellite instability (MSI) status could have been valuable, since the mutational status has been shown to predict recurrence patterns after liver resection [42, 43], but the mutational status was not routinely analyzed at Helsinki University Hospital prior to 2013 and the MSI not prior to 2018.****”*

2) From a statistical standpoint, was Bonferroni correction applied to adjust for multiple testing, given the authors evaluation of outcomes categorized as: 0 elevated/1 elevated/ 2 elevated/3 elevated/ 4 elevated/5 elevated and then subsequently as: 0-1 elevated/2-5 elevated. Also, were other categorizations evaluated as well (i.e 0-2 elevated/3-5 elevated) and if so, it further strengthens the role for application of Bonferroni correction.

*Bonferroni correction was not taken into account, and thus the results may be due to chance. The large number of patients included in the material increases the statistical validity of the results, but as stated in our Discussion, we acknowledge the statistical uncertainty surrounding our findings and state that our results will need external validation, whereby an independent cohort can show the same results in order to attach certainty to the results we obtain. We have tried to investigate our cohort using different approaches. The only comparison of number of elevated markers was 0–1 vs. 2–5, which can be seen as an exploration of testing each number of elevated markers against zero elevated markers. No other combinations were tested, which is why the use of Bonferroni correction is not justified by this particular aspect. We have updated the sections “Results” and “Discussion” mentioning the additional testing of 0–1 vs. 2–5 elevated markers, thus attaching less certainty to this finding, and we hope that this will alleviate the Reviewer’s concern.*

3) How was 3 months postop chosen as the optimal time point for this analysis? Was there subsequent serial draws at other time points as part of the study as well?

*We thank the Reviewer for this question. We chose that time point, because we hypothesized that 3 months after liver resection, the possible postoperative complications would have resolved and the liver tissue would have regenerated, and thus, the surgical trauma would not affect the tumor marker levels anymore. We also estimated that earlier increase in the biomarker levels would most probably be due to either residual disease or surgical trauma and not recurrence.*

*Serum samples were also drawn one week after resection, but those samples were not included in this study. Their prognostic value would have been controversial, since the levels of the inflammatory biomarkers rise postoperatively merely due to surgical trauma (Shantha Kumara HM et al. World J Gastrointest Oncol. 2016;8(8):607-14.).*

4) Given increasing data evaluating early detection of disease recurrence with liquid biopsies, the authors should mention it as emerging technologies that are challenging the prognostic significance of traditional biomarkers like CEA/CA 19-9.

*We thank the Reviewer for this observation. We have now mentioned this in the section “Introduction” as follows:*

*“****The early detection of cancer with emerging technologies, such as liquid biopsies that consist of circulating tumor cells, DNA, and exosomes in the peripheral blood, is under active investigation [22]{Normanno, 2018 #119}. However, protein biomarkers are at present an integral part of clinical work and may be more easily accessed.****”*

5) Interesting that CA 19-9 was the only significant biomarker on MVA in the preoperative setting for RFS and only CEA and CA 19-9 were significant in the postoperative setting on MVA. This suggests that perhaps CEA and CA 19-9 (known prognostic factors) are what is driving the significance of the 2-5 biomarkers, and not YKL-40, IL-6 and CRP. Where any of the other clinical variables on MVA significant?

*To respond to the Reviewer’s concerns in this aspect, we have explored this further by studying three groups of patients in an additional explorative analysis, namely patients with zero elevated markers, patients with elevated CEA and/or CA19-9, and patients with elevated YKL-40, IL-6 and/or CRP. The results of this analysis are included as the S6 Table in the Supporting Information files. They showed that patients with elevated CEA and/or CA19-9 postoperatively had a significantly higher risk of relapse or death, while elevated YKL-40, IL-6, and/or CRP was not significantly associated with increased risk of relapse or death in any instance. We have already analyzed the biomarkers together in a multivariate analysis, although on a continuous scale, which showed that mainly CEA and CA19-9 were the drivers behind the negative prognostic value of having several elevated biomarkers, although YKL-40 was also associated with a negative prognostic outcome as a preoperative marker in relation to OS. The sections “Results” and “Discussion” have been updated with comments on these explorative findings.*

6) Table 1 as current presented is confusing and hard to follow. I would suggest that the authors remove outcome data from the Table 1 (alive/dead), and then present the biomarker data in the more concise and readable manner. Also please add R0/R1/R2 rates to Table. 1

*We have now edited the Table 1 according to the Reviewer’s suggestions. To present the biomarker data in a more readable manner, we separated them from the patient characteristics and created a new Table 2.*

*However, we decided to keep the outcome data included in Table 1, since we find that the information helps the readers to get a better overall understanding of the patient material.*

7) On page 10, line 201: Please edit to include "WERE".....Ca 19-9 (HR 1.08) WERE associated

*We have corrected the sentence according to the Reviewer’s suggestion.*

8) Similalry, on page 12: Please edit to include "WERE"....CA 19-9 (HR 1.12) WERE associated...

*We have corrected the sentence according to the Reviewer’s suggestion.*
