# Supplementary material for: Elevated serum YKL-40, IL-6, CRP, CEA, and CA19-9 combined as a prognostic biomarker panel after resection of colorectal liver metastases
Source: PLoS One. 2020 Aug 5;15(8):e0236569. doi: 10.1371/journal.pone.0236569 (PMC7406016; doi:10.1371/journal.pone.0236569)
Supplement: Attachment — Submitted filename: Response to Reviewers (re-revisions).docx [file pone.0236569.s011.docx]

PLOS ONE

Date: June 1^st^, 2020

PONE-D-19-31995R1
Elevated serum YKL-40, IL-6, CRP, CEA, and CA19-9 combined as a prognostic biomarker panel after resection of colorectal liver metastases

**Responses to the comments of the Academic Editor and the Reviewers**

We thank the Reviewers for accurate comments and constructive criticism. We have revised the manuscript according to the suggestions, and responses to the comments and questions are shown below. Our responses are written in red italics. We strongly feel that these changes have improved the manuscript.

**Additional Editor Comments (if provided):**

The reviewers have identified some concerns about the statistical approach. Please address these along with their other concerns if you wish to revise the manuscript again.

**Reviewers' comments:**

Reviewer's Responses to Questions

**Comments to the Author**

1. If the authors have adequately addressed your comments raised in a previous round of review and you feel that this manuscript is now acceptable for publication, you may indicate that here to bypass the “Comments to the Author” section, enter your conflict of interest statement in the “Confidential to Editor” section, and submit your "Accept" recommendation.

Reviewer #1: All comments have been addressed

Reviewer #2: (No Response)

Reviewer #3: (No Response)

2. Is the manuscript technically sound, and do the data support the conclusions?

The manuscript must describe a technically sound piece of scientific research with data that supports the conclusions. Experiments must have been conducted rigorously, with appropriate controls, replication, and sample sizes. The conclusions must be drawn appropriately based on the data presented.

Reviewer #1: Yes

Reviewer #2: Partly

Reviewer #3: Partly

3. Has the statistical analysis been performed appropriately and rigorously?

Reviewer #1: Yes

Reviewer #2: I Don't Know

Reviewer #3: No

4. Have the authors made all data underlying the findings in their manuscript fully available?

The PLOS Data policy requires authors to make all data underlying the findings described in their manuscript fully available without restriction, with rare exception (please refer to the Data Availability Statement in the manuscript PDF file). The data should be provided as part of the manuscript or its supporting information, or deposited to a public repository. For example, in addition to summary statistics, the data points behind means, medians and variance measures should be available. If there are restrictions on publicly sharing data—e.g. participant privacy or use of data from a third party—those must be specified.

Reviewer #1: Yes

Reviewer #2: Yes

Reviewer #3: Yes

5. Is the manuscript presented in an intelligible fashion and written in standard English?

PLOS ONE does not copyedit accepted manuscripts, so the language in submitted articles must be clear, correct, and unambiguous. Any typographical or grammatical errors should be corrected at revision, so please note any specific errors here.

Reviewer #1: Yes

Reviewer #2: Yes

Reviewer #3: Yes

**6. Review Comments to the Author**

Please use the space provided to explain your answers to the questions above. You may also include additional comments for the author, including concerns about dual publication, research ethics, or publication ethics. (Please upload your review as an attachment if it exceeds 20,000 characters)

Reviewer #1: The authors have addressed all comments with the submitted revision, and limitations to the generalizability to the findings have been adequately detailed in the Discussion section.

Reviewer #2: Overall, I appreciate the reviewer's responses to commentary.

The one persistent issue I would raise is why the authors place emphasis and suggest benefit of IL-6 and CRP when I am not sure the data shows this. When you tease out the results, it appears that CEA and CA 19-9 are driving the prognostic significance. CRP is not significant on any UVA or MVA either in preop or postop. IL-6 and YLK-40 are significant on UVA, but not MVA. And the authors even perform a requested analysis at reviewers' request that demonstrates: "We have now explored this further by studying three groups of patients in an additional explorative analysis, namely patients with zero elevated markers, patients with CEA and/or CA19-9 elevated, and patients with YKL-40, IL-6 and/or CRP elevated. The results of this analysis are included as S6 Table in the Supporting Information files. They showed that patients with elevated CEA and/or CA19-9 elevated postoperatively had a significantly higher risk of relapse or death, while elevated YKL-40, IL-6, and/or CRP was not significantly associated with increased risk of relapse or death in any instance."

All in all, I somehow have a hard time wrapping my head around the conclusion that the "combined prognostic value of all 5 markers has benefit" as the results of the study do not support these findings.

*- We thank the Reviewer for again taking the time to review our work in this second revision. As for the above-mentioned concern, we agree to some extent with the Reviewer, and we are glad that the other changes made in the latest revision were of satisfactory quality. We have addressed the Reviewer’s concern by changing our wording to be less reflective of a perceived benefit of our biomarker panel as it, indeed, cannot be said that it unequivocally has benefit (please see lines 57–58 in the abstract, lines 357–359, and line 378 in the present revised manuscript).*

Reviewer #3: The manuscript entitled ‘Elevated serum YKL-40, IL-6, CRP, CEA, and CA19-9 combined as a prognostic biomarker panel after resection of colorectal liver metastases’ with the aim to examine their associations with relapse-free survival and overall survival in combination with serum C-reactive protein (CRP), carcinoembryonic antigen (CEA), and carbohydrate antigen 19-9 (CA19-9) in patients with colorectal liver metastases.

This is an interesting study, however, the manuscript requires further improvement.

Comments

Abstract, all presentation of CI95% to be written as 95% CI. This apply throughout the manuscript i.e Page 10 Line 195, Page 24 Table 3, Page 27 Table 4, Supplementary Table 5A, 5B, 5C and 5D, 6.

*- We have made the corrections according to the Reviewer’s suggestion.*

The abstract to be labelled with heading Background, Methods, Results, Conclusion.

*- We have corrected the Abstract according to the Reviewer’s suggestion.*

Materials and methods
It would be good to include a study flowchart.

*- We have now included in the manuscript a study flow diagram as a Supplementary Figure 1.*

There was no sample size calculation or power of study from the sample size used was discussed in the manuscript.

*- We did not perform a post-hoc power calculation nor do we find it necessary. Most post-hoc power estimations can be easily biased. As the present study was not planned prior to the collection of samples, an a priori sample size calculation was not needed for this specific study. Power estimations are essential when applying for ethics approval, but this is done at a much earlier stage in planning.*

Page 7 Line 151. P95% (subscript 95%) to be written as P95%.

*- The correction is made according to the Reviewer’s suggestion.*

Statistical analyses
Page 7 Line 155-158, the sentence on the function of statistical tests (Wilcoxon signed-rank test and Spearman’s rank correlation coefficient) incomplete and requires improvement.

*- We thank the Reviewer for bringing our attention to this issue, although we do not appreciate in the same way what is incomplete about the sentence. We explicitly state that these tests were used to test for pre- and postoperative differences in serum values, and we use a Spearman’s rank test for testing for correlations. Later in the manuscript we mention the results of correlations and p-values for differences in the serum values pre- and postoperatively. If the Reviewer continues to find the sentence erroneous, please direct us to what is specifically incomplete about the sentence and the specific improvements needed.*

Page 7 Line 163, the reason to use LOD/2 as one of the imputation methods for values below the LOD to be stated.

*- The reason is that we believed the most correct value was a non-zero value, and chose a pragmatic approach to dealing with “missing” values. Please see the lines 165–167.*

Page 7 Line 173, full name for AUC to be stated.

*- The correction is made according to the Reviewer’s suggestion.*

It would be good to include a brief description on the variables coding which were used in the analysis.

*- We only coded the variable number of elevated markers by simply counting the number of elevated markers a patient had according to the already mentioned cut points. We specifically state the investigated cut points in the Methods section, and we hope that this is sufficient for the Reviewer.*

Results
Page 21 Table 1, the title is too short. Decimal points for percentages to be standardized.

*- We have now standardized the percentages and lengthened the title.*

Page 21 Table 1 and Page 22 Table 2 and text, the word range to be replaced or to be denoted as min-max.

*- The word “range” is now replaced by “min.–max.”.*

Page 22 Table 2, symbol N to be replaced with n. N to be used for overall/Total sample size. Total sample size for each marker at pre and post operative to be stated.

*- The corrections are made according to the Reviewer’s suggestion.*

Page 10 Line 202, 205, 205, 207, 210 the word pre operative or post operatively to be stated for YKL-40, CEA, CEA, CA19-9, CEA.

*- The section is now edited according to the Reviewer’s suggestion.*

Page 24 Table 3, Page 27 Table 4, the 95%CI for the 5 elevated are wide. Perhaps data for 4 elevated and 5 elevated could be merged as one.

*- These confidence intervals are wide since very few patients have all five markers elevated. We do not believe that rearranging groups according to the confidence intervals they present in the analyses is a valid method of defining groups, and it could easily lead to statistical cosmetic improvement. We hope that the readers will interpret the confidence intervals warily and accordingly, and we ourselves do not put emphasis in these findings.*

Page 25 & 26 Fig 1 & 2 and Fig 3 & 4 title, the number elevated to be stated in order to differentiate the titles.

*- The corrections are made according to the Reviewer’s suggestion.*

Supplementary Table 5A-5D & Table 6, (0) to be omitted from Synchronous (0).

*- The corrections are made according to the Reviewer’s suggestion.*

Model fit information for the multivariate analysis to be stated.

*- We state in the section “Methods” that the “Proportional hazards assumption was evaluated by testing for trends in the scaled Schoenfeld residuals” (lines 174–175). This should suffice for fitting of the models as no consensus exists on the correct model fit number to compare Cox regression models.*

The statement/reason on 'Bonferroni correction' to be clearly stated in the discussion.

*- We have now edited the section “Discussion” according to the suggestion (lines 364–365).*

References did not conform to the journal format.

*- We use the EndNote style format provided by the journal itself. If any issues should arise later in the editorial process, we trust it that the editorial staff will correct any mistakes in typesetting and direct us to the correct format of references.*

7. PLOS authors have the option to publish the peer review history of their article. If published, this will include your full peer review and any attached files.

If you choose “no”, your identity will remain anonymous but your review may still be made public.

**Do you want your identity to be public for this peer review?** For information about this choice, including consent withdrawal, please see our Privacy Policy.

Reviewer #1: No

Reviewer #2: No

Reviewer #3: No
