# Supplementary material for: Elevated serum YKL-40, IL-6, CRP, CEA, and CA19-9 combined as a prognostic biomarker panel after resection of colorectal liver metastases
Source: PLoS One. 2020 Aug 5;15(8):e0236569. doi: 10.1371/journal.pone.0236569 (PMC7406016; doi:10.1371/journal.pone.0236569)
Supplement: Attachment — Submitted filename: Response to Reviewers.docx [file pone.0236569.s012.docx]

PLOS ONE

Date: July 6^th^, 2020

PONE-D-19-31995R2
Elevated serum YKL-40, IL-6, CRP, CEA, and CA19-9 combined as a prognostic biomarker panel after resection of colorectal liver metastases

**Responses to the comments of the Academic Editor and the Reviewers**

We thank the Reviewer #1 for the accurate comment and constructive criticism. We have revised the manuscript according to the suggestion, and our response is shown below written in red italics.

**Additional Editor Comments (if provided):**

The manuscript is acceptable with one minor revision in the Discussion as requested by Reviewer 1. Please address this point in the Discussion and resubmit.

**Reviewers' comments:**

6. Review Comments to the Author

Reviewer #1: I think that the authors have made their case about the five biomarkers here. It still remains very unclear the clinical practicality of these findings, as I could not envision these ultimately making it to the clinic for routine use, especially in the emerging era of ctDNA technologies, which are more sensitive and specific than the biomarker combinations detailed here. I think that acknowledgement of the ctDNA data for the metastatic CRC resections as a prognostic biomarker could help further put the authors' conclusions into a more accurate context. I think it is more likely that ctDNA would be the more feasible route for personalizing management of perioperative therapies. I would recommend the authors updating the discussion to include some of the ctDNA findings.

*- We thank the Reviewer for this observation. We have now edited the section “Discussion” and pondered the prognostic value of the biomarkers investigated in this study in relation to the new emerging technologies, such as liquid biopsies, as follows (lines 361–369):*

***“It is probable that new technologies, liquid biopsies in particular, will be applied to clinical use quite shortly. They have potential in the detection and management of CRC, as they offer a non-invasive method for early detection of cancer, prognostic and predictive information, monitoring of treatment response, and identification of minimal residual disease [41]. In conjunction with resection of CRLM, elevated pre- [42] and postoperative [43] ctDNA levels have shown high concordance with relapse in ctDNA-positive patients. However, it seems unlikely that liquid biopsies could provide information on inflammatory responses, which affect the prognosis of CRC. Thus, the inflammatory biomarkers have an important role also in this era of advances in these new technologies.”***

*41.	Yamada T, Matsuda A, Koizumi M, Shinji S, Takahashi G, Iwai T, et al. Liquid Biopsy for the Management of Patients with Colorectal Cancer. Digestion. 2019;99(1):39-45. doi: 10.1159/000494411.*

*42.	He Y, Ma X, Chen K, Liu F, Cai S, Han-Zhang H, et al. Perioperative Circulating Tumor DNA in Colorectal Liver Metastases: Concordance with Metastatic Tissue and Predictive Value for Tumor Burden and Prognosis. Cancer Manag Res. 2020;12:1621-30. doi: 10.2147/CMAR.S240869. PubMed PMID: 32184665.*

*43.	Benešová L, Hálková T, Ptáčková R, Semyakina A, Menclová K, Pudil J, et al. Significance of postoperative follow-up of patients with metastatic colorectal cancer using circulating tumor DNA. World J Gastroenterol. 2019;25(48):6939-48. doi: 10.3748/wjg.v25.i48.6939. PubMed PMID: 31908397.*

Reviewer #2: Thank you very much for the responses to my concerns. The authors have adequately addressed my concerns.

Reviewer #3: (No Response)
